# Interpreter Availability for Asylum Seekers/Non-English Speakers in Mental Health Acute Care Setting at Mersey Care NHS Foundation Trust

**DOI:** 10.1192/bjo.2025.10645

**Published:** 2025-06-20

**Authors:** Mahmoud Odeh, Haruna Ngada, Ranjan Baruah, Shazlina Shahul

**Affiliations:** Mersey Care NHS Foundation Trust, Liverpool, United Kingdom

## Abstract

**Aims:** The aim was to examine if the communication needs of asylum seekers/non-English speakers who were admitted to Acute Care Settings were addressed on admission and during the first ward round to ensure compliance with the accessibility standard act that is mandated by the whole NHS and to ensure compliance with Mersey Care NHS Foundation Trust (MCFT) Policy.

The Accessible Information Standard (AIS) SCCI1605, mandated by NHS England in 2015, is a legal duty placed on all organizations providing NHS or adult social care. Communication and/or information needs must be identified at registration/upon first contact with the service or as soon as is practicable thereafter.

One of the fundamental principles of AIS is that patients, service users, carers and parents should be asked to self-define their information and/or communication support needs. It is these needs (not their disability) which should be recorded. This initial question may be asked over the telephone, face to face at a reception desk, as part of a registration or admission form or through an alternative process and thereafter proactively and opportunistically.

**Methods:** This was a retrospective audit.

The inclusion criteria were Asylum seeker/non-English-speakers who cannot speak, understand and comprehend English. The exclusion criteria were Asylum seeker/non-English-speakers who can speak, understand and comprehend English.

Data from 24 patients during a 12-month period was collected and collated.

**Results:** Communication needs of asylum seekers/non-English speakers who don’t or have limited English speaking and understanding capabilities were recognised and documented clearly in the first 24 hours of their admission in 83%.

79% asylum seekers/non-English speakers who do not or have limited English speaking and understanding capabilities had interpreting services involved when important decisions regarding their care is being made during the first ward round.

Of the 24 cases audited, 14 had services involved face to face, phone, virtually, or other. Of the 10 that did not, 5 had a reason documented.

**Conclusion:** There is still room for improvement to meet the communication needs for the audited cohort.

Audit Team would put recommendations to MCFT to consider:

Amending the admission proforma to include a section for identifying and documenting any specific communication needs of the patient.

Incorporating a communication needs alert system for all patients to ensure staff are vigilant to response.

A bigger sample size over a longer time frame including multiple acute care settings in the trust would be planned.

